# Welcome to *Journal of Ovarian Research*

**DOI:** 10.1186/1757-2215-1-1

**Published:** 2008-10-01

**Authors:** Stefano Palomba, David T Curiel, Sham S Kakar, Fumikazu Kotsuji

**Affiliations:** 1 University "Magna Graecia" of Catanzaro, Viale Europa, Catanzaro, Italy; 2 Gene Therapy Center, University of Alabama at Birmingham, Birmingham AL, USA; 3 University of Louisville, Louisville KY, USA; 4 University of Fukui, Fukui, Japan

In the last few years interest in the normal physiology and disease pathology of the ovary has increased. A search of bibliographical records carried out without limits for language and type of article, inserting only "ovary" as a key word, produced a total number of 101,414 published articles, of which 3,040 were published last year.

Such studies have been disseminated to a diffuse subset of journals, which handle studies related to the ovary but are not specialized on the subject. In fact, presently, there is no journal dedicated to covering the extensive research carried out into ovarian functions and possible interactions with other systems regulating the correct function of the female organism. On this basis, it has been difficult to gain a full view of the overall progress and to appreciate the associations within the fields that could predicate productive linkages. As for the case of five blind men and the elephant, a larger understanding of the field's evolution has remained elusive owing to focused studies, reported diversely, which ignored the fields' expansion in its entirety.

The need to launch a new journal having ovarian biology/pathology (gender physiopathology) as its only field of interest was a consequence of these considerations. Accordingly, *Journal of Ovarian Research *aims to face open questions in ovarian biology/pathology using experimental, translational and clinical approaches.

The journal will certainly cover research relevant to the, already well-known, reproductive functions of the ovary. In addition, in the last few years, interesting research has been carried out with the aim to investigate the function of the ovary as an endocrine organ; one must consider the intricate signalling networks at ovary level mediated by various paracrine and endocrine factors regulating follicular growth and hormone secretion. Studies in this area will be welcomed by the journal.

Research in the area of ovarian cancer is also of great interest. In demonstration of increasing interest on the subject is the creation of ultra-specialised centres for diagnosis and treatment of ovarian cancer. Due to the specific familiar and genetic traits of ovarian cancer, various studies have been conducted on possible risk factors and innovative therapies. In particular, in the last few years numerous studies have been carried out to investigate the role of benignant pathologies in the risk of ovarian cancer, such as endometriosis and polycystic ovarian syndrome. Some studies seem to indicate hormonal therapy for menopause (hormone replacement therapy, HRT) and drugs for ovulation induction (clomiphene citrate and gonadotropins) as possible factors, which increase, through unclear mechanisms, the risk of ovarian cancer. On the other hand, among the non-contraceptive benefits of estroprogestin compounds, an oncological protective effect on ovarian tissues was acknowledged.

Lastly, but no less important, in the last few years various experimental *in-vitro *and animal model studies have been published on topics such as molecular intraovarian regulation, and regulation from other organs; possible onco-genetic mechanisms; drug effects; the possibility of oocyte cryoconservation; autologous and heterologous ovarian tissue transplant and the use of staminal cells. All these hypotheses and experimental results, which presently seem futuristic, and, in some cases, peculiar, could have, in the following years, a remarkable clinical implication.

*Journal of Ovarian Research *will therefore provide an online open access home for research that provides new insights into ovarian functions as well as prevention and treatment of diseases afflicting the organ. The Editorial Board is composed of international experts from various scientific fields. Authors should submit their manuscripts directly online via the journal's website. Manuscripts will be reviewed by expert referees and we aim to give a first decision within six weeks from submission. Articles accepted for publication will be published immediately online, ensuring a fast and efficient process.

Obviously, we are still at the beginning of this ambitious scientific-editorial enterprise, and we recognize that with the profusion of new journals, it may be an inauspicious time for launching a new enterprise of such specialized scope. However we hope the journal will become a high quality forum for researchers in the field and will be indexed rapidly, reaching an adequate impact factor in the next few years. Moreover, we hope that the journal will be of support to international scientific societies offering ample availability to widespread congress proceedings, guidelines and integrated strategies in research.

Concluding, we hope that this new journal becomes a stable point of reference for researchers in the field of ovarian research. A Japanese proverb holds that "One's most important task in life is to become a useful person". The task of *Journal of Ovarian Research *therefore is to become a useful vehicle for our community of investigators.

